# Dichloridobis(phenyl 2-pyridyl ketone oxime)nickel(II) acetone solvate

**DOI:** 10.1107/S1600536808043961

**Published:** 2009-01-08

**Authors:** Jing-Zhou Yin, Guang-Xiang Liu

**Affiliations:** a Jiangsu Key Laboratory for Chemistry of Low-dimensional Materials, Department of Chemistry, Huaiyin Teachers College, Huai’an 223300, People’s Republic of China; b Anhui Key Laboratory of Functional Coordination Compounds, School of Chemistry and Chemical Engineering, Anqing Normal University, Anqing 246003, People’s Republic of China

## Abstract

The Ni atom in the title compound, [NiCl_2_(C_12_H_10_N_2_O)_2_]·C_3_H_6_O, adopts a distorted octa­hedral geometry, being ligated by four N atoms from two different phenyl 2-pyridyl ketone oxime ligands and two Cl atoms. In the crystal structure, inter­molecular O—H⋯Cl hydrogen bonds link the mol­ecules into a chain structure along [010]. There is a π–π contact between the pyridine rings [centroid–centroid distance = 3.824 (5) Å].

## Related literature

For related structures, see: Korpi *et al.* (2005 ▶); Pearse *et al.* (1989 ▶); Afrati *et al.* (2005 ▶); Stamatatos *et al.* (2006 ▶); Papatriantafyllopoulou *et al.* (2007 ▶).
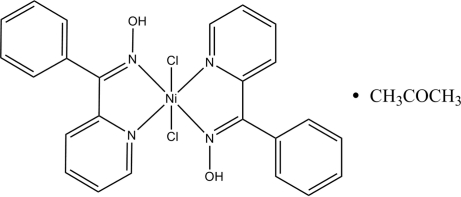


## Experimental

### 

#### Crystal data

[NiCl_2_(C_12_H_10_N_2_O)_2_]·C_3_H_6_O
*M* *_r_* = 584.13
Triclinic, 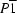

*a* = 9.0367 (11) Å
*b* = 12.9142 (16) Å
*c* = 13.0664 (16) Å
α = 105.4390 (10)°
β = 92.232 (2)°
γ = 108.183 (2)°
*V* = 1384.0 (3) Å^3^
*Z* = 2
Mo *K*α radiation
μ = 0.93 mm^−1^
*T* = 296 (2) K
0.22 × 0.18 × 0.16 mm

#### Data collection

Bruker SMART APEX CCD area-detector diffractometer
Absorption correction: multi-scan (*SADABS*; Bruker, 2000 ▶) *T*_min_ = 0.822, *T*_max_ = 0.866
6839 measured reflections
4761 independent reflections
4002 reflections with *I* > 2σ(*I*)
*R*_int_ = 0.021

#### Refinement

*R*[*F* ^2^ > 2σ(*F* ^2^)] = 0.032
*wR*(*F* ^2^) = 0.089
*S* = 1.05
4761 reflections
338 parameters
H-atom parameters constrained
Δρ_max_ = 0.26 e Å^−3^
Δρ_min_ = −0.39 e Å^−3^

### 

Data collection: *SMART* (Bruker, 2000 ▶); cell refinement: *SAINT* (Bruker, 2000 ▶); data reduction: *SAINT*; program(s) used to solve structure: *SHELXS97* (Sheldrick, 2008 ▶); program(s) used to refine structure: *SHELXL97* (Sheldrick, 2008 ▶); molecular graphics: *SHELXTL* (Sheldrick, 2008 ▶); software used to prepare material for publication: *SHELXTL*.

## Supplementary Material

Crystal structure: contains datablocks I, global. DOI: 10.1107/S1600536808043961/at2700sup1.cif

Structure factors: contains datablocks I. DOI: 10.1107/S1600536808043961/at2700Isup2.hkl

Additional supplementary materials:  crystallographic information; 3D view; checkCIF report

## Figures and Tables

**Table 1:** Selected geometric parameters (Å, °)

Ni1—N3	2.0344 (18)
Ni1—N1	2.0418 (18)
Ni1—N4	2.0879 (17)
Ni1—N2	2.1188 (17)
Ni1—Cl1	2.3944 (6)
Ni1—Cl2	2.4153 (7)

**Table 2:** Hydrogen-bond geometry (Å, °)

*D*—H⋯*A*	*D*—H	H⋯*A*	*D*⋯*A*	*D*—H⋯*A*
O2—H2⋯Cl2	0.82	2.27	2.9582 (18)	142
O1—H1⋯Cl1^i^	0.82	2.91	3.4612 (16)	127
O1—H1⋯Cl1	0.82	2.37	3.0542 (16)	141

Symmetry code: (i) 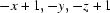.

**Fig. 1.**
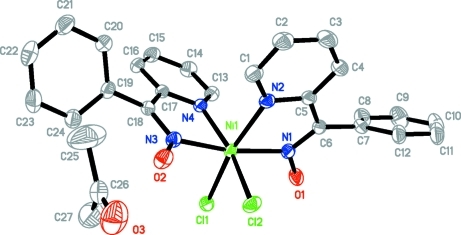
The molecular structure of the title compound, with atom labels and 30% probability displacement ellipsoids. The solvent molecule and H atoms have been omitted for clarity.

**Fig. 2.**
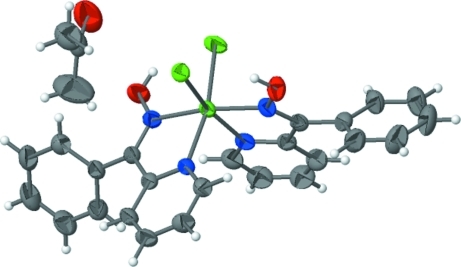
A packing diagram of the title compound. Hydrogen bonds are shown as dashed lines.

## supplementary crystallographic information

### Comment

Pyridine-2-carbaldehyde oxime ligands usually bind to metals in a bidentate fashion, either chelating one metal center or bridging two metals. Their complexes find application in diverse areas such as functional supramolecular design, magnetic materials and catalysis (Korpi *et al.*, 2005; Pearse *et al.*, 1989; Afrati *et al.*, 2005; Stamatatos *et al.*, 2006). The title compound is a new nickel complex from the reaction of NiCl_2_ with phenyl-2-pyridyl ketone oxime (ppo). The compound consists of two *N*,*N*-chelating ligands and two chloride anion. The two ppo ligands are coordinated to Ni to form two five-membered NiC_2_N_2_ rings. The central Ni atom adopts a distorted octahedral geometry (Fig. 1), which are ligated by four N atoms from two different phenyl-2-pyridyl ketone oxime ligand and two Cl atoms. The bond distances Ni—N and Ni—Cl are in the expected ranges of 2.0344 (18)–2.1188 (17) and 2.3944 (6)–2.4153 (7) Å, respectively, and the coordination angles around Ni atom are in the range 76.84 (7)–170.18 (7)°, which are in agreement with the literature values (Papatriantafyllopoulou *et al.*, 2007). In the crystal structure, intermolecular O—H···Cl hydrogen bonds link the molecules into one-dimensional chain structure (Table 2). There is a π–π contact between the pyridine rings, and the distance of centroid to centroid is 3.824 (5) Å.

### Experimental

A colourless solution of phenyl-2-pyridyl ketone oxime (0.197 g, 1.00 mmol) in acetone (10 ml) was slowly added to a slurry of LiOH.H_2_O (0.042 g, 1.00 mmol) in MeOH (5 ml); the hydroxide soon dissolved. The solution was then added to a slurry of NiCl2.6H2O (0.297 g, 1.00 mmol) in MeOH (10 ml) and the resulting green solution was stirred for 1 h at room temperature. A small quantity of undissolved material was removed by filtration. The filtrate was allowed to stand undisturbed in a closed flask for a period of 4–5 d. Dark cyan crystals appeared which were collected by filtration, washed with cold MeOH (1 ml) and ice-cold Et_2_O (2 ml), and dried in air [Yield: 52%].

### Refinement

All H atoms were placed in calculated positions, with O—H = 0.82 Å, *U*_iso_(H) = 1.5*U*_eq_(O), C—H = 0.93 Å, *U*_iso_(H) = 1.2*U*_eq_(C) for aromatic and C—H = 0.96 Å, *U*_iso_(H) = 1.5*U*_eq_(C) for CH_3_ atoms.

### Crystal data

**Table:**

[NiCl_2_(C_12_H_10_N_2_O)_2_]·C_3_H_6_O	*Z* = 2
*M**_r_* = 584.13	*F*(000) = 604
Triclinic, *P*1	*D*_x_ = 1.402 Mg m^−^^3^
Hall symbol: -P 1	Mo *K*α radiation, λ = 0.71073 Å
*a* = 9.0367 (11) Å	Cell parameters from 3432 reflections
*b* = 12.9142 (16) Å	θ = 2.4–27.6°
*c* = 13.0664 (16) Å	µ = 0.93 mm^−^^1^
α = 105.439 (1)°	*T* = 296 K
β = 92.232 (2)°	Block, dark cyan
γ = 108.183 (2)°	0.22 × 0.18 × 0.16 mm
*V* = 1384.0 (3) Å^3^	

### Data collection

**Table:**

Bruker SMART APEX CCD area-detector diffractometer	4761 independent reflections
Radiation source: sealed tube	4002 reflections with *I* > 2σ(*I*)
graphite	*R*_int_ = 0.021
φ and ω scans	θ_max_ = 25.0°, θ_min_ = 1.6°
Absorption correction: multi-scan (*SADABS*; Bruker, 2000)	*h* = −10→6
*T*_min_ = 0.822, *T*_max_ = 0.866	*k* = −10→15
6839 measured reflections	*l* = −15→15

### Refinement

**Table:**

Refinement on *F*^2^	Primary atom site location: structure-invariant direct methods
Least-squares matrix: full	Secondary atom site location: difference Fourier map
*R*[*F*^2^ > 2σ(*F*^2^)] = 0.032	Hydrogen site location: inferred from neighbouring sites
*wR*(*F*^2^) = 0.089	H-atom parameters constrained
*S* = 1.05	*w* = 1/[σ^2^(*F*_o_^2^) + (0.0472*P*)^2^] where *P* = (*F*_o_^2^ + 2*F*_c_^2^)/3
4761 reflections	(Δ/σ)_max_ < 0.001
338 parameters	Δρ_max_ = 0.26 e Å^−^^3^
0 restraints	Δρ_min_ = −0.39 e Å^−^^3^

### Special details

**Table:**

Geometry. All e.s.d.'s (except the e.s.d. in the dihedral angle between two l.s. planes) are estimated using the full covariance matrix. The cell e.s.d.'s are taken into account individually in the estimation of e.s.d.'s in distances, angles and torsion angles; correlations between e.s.d.'s in cell parameters are only used when they are defined by crystal symmetry. An approximate (isotropic) treatment of cell e.s.d.'s is used for estimating e.s.d.'s involving l.s. planes.
Refinement. H atoms were positioned geometrically, with C—H = 0.93 and 0.96 Å for aromatic and methyl H atoms, respectively, and constrained to ride on their parent atoms, with *U*_iso_(H) = xUeq(C), where *x* = 1.5 for methyl H and *x* = 1.2 for aromatic H atoms. The highest peak is located 1.10 Å from atom Cl1.

### Fractional atomic coordinates and isotropic or equivalent isotropic displacement parameters (Å^2^)

**Table:**

	*x*	*y*	*z*	*U*_iso_*/*U*_eq_	
Ni1	0.34978 (3)	0.11803 (2)	0.295182 (19)	0.03252 (11)	
Cl1	0.43421 (6)	0.11649 (4)	0.47059 (4)	0.03838 (15)	
Cl2	0.61388 (7)	0.19661 (5)	0.25662 (5)	0.04710 (16)	
O1	0.3728 (2)	−0.11019 (13)	0.29824 (12)	0.0508 (4)	
H1	0.4028	−0.0694	0.3602	0.076*	
O2	0.45576 (19)	0.36884 (13)	0.31744 (15)	0.0519 (4)	
H2	0.5345	0.3509	0.3069	0.078*	
O3	0.8381 (3)	0.7143 (2)	0.2311 (2)	0.0984 (8)	
N1	0.3351 (2)	−0.04856 (15)	0.23669 (13)	0.0373 (4)	
N2	0.2672 (2)	0.07731 (16)	0.13055 (14)	0.0380 (4)	
N3	0.3367 (2)	0.27794 (14)	0.33148 (14)	0.0367 (4)	
N4	0.1224 (2)	0.09447 (14)	0.33492 (13)	0.0327 (4)	
C1	0.2320 (3)	0.1443 (2)	0.07928 (19)	0.0488 (6)	
H1A	0.2365	0.2169	0.1190	0.059*	
C2	0.1888 (3)	0.1112 (2)	−0.0308 (2)	0.0579 (7)	
H2A	0.1652	0.1604	−0.0643	0.070*	
C3	0.1820 (3)	0.0046 (3)	−0.0884 (2)	0.0629 (8)	
H3	0.1555	−0.0193	−0.1624	0.076*	
C4	0.2143 (3)	−0.0681 (2)	−0.03703 (18)	0.0527 (6)	
H4	0.2073	−0.1416	−0.0756	0.063*	
C5	0.2575 (3)	−0.02919 (19)	0.07348 (17)	0.0395 (5)	
C6	0.2956 (2)	−0.10087 (18)	0.13567 (16)	0.0368 (5)	
C7	0.2878 (3)	−0.21931 (19)	0.08362 (17)	0.0416 (5)	
C8	0.1900 (3)	−0.3086 (2)	0.1158 (2)	0.0586 (7)	
H8	0.1293	−0.2939	0.1701	0.070*	
C9	0.1835 (4)	−0.4189 (2)	0.0668 (2)	0.0736 (9)	
H9	0.1187	−0.4784	0.0887	0.088*	
C10	0.2724 (4)	−0.4419 (2)	−0.0146 (2)	0.0737 (9)	
H10	0.2667	−0.5166	−0.0476	0.088*	
C11	0.3686 (4)	−0.3545 (2)	−0.0462 (2)	0.0672 (8)	
H11	0.4291	−0.3699	−0.1006	0.081*	
C12	0.3768 (3)	−0.2433 (2)	0.00211 (18)	0.0501 (6)	
H12	0.4424	−0.1843	−0.0202	0.060*	
C13	0.0213 (3)	0.00010 (18)	0.34823 (17)	0.0396 (5)	
H13	0.0474	−0.0661	0.3316	0.047*	
C14	−0.1203 (3)	−0.0034 (2)	0.38561 (19)	0.0464 (6)	
H14	−0.1876	−0.0707	0.3942	0.056*	
C15	−0.1606 (3)	0.0934 (2)	0.40997 (18)	0.0463 (6)	
H15	−0.2558	0.0926	0.4349	0.056*	
C16	−0.0576 (3)	0.1920 (2)	0.39698 (17)	0.0413 (5)	
H16	−0.0826	0.2587	0.4129	0.050*	
C17	0.0833 (2)	0.19055 (17)	0.36005 (15)	0.0322 (5)	
C18	0.2045 (2)	0.29384 (17)	0.34868 (16)	0.0348 (5)	
C19	0.1721 (3)	0.40061 (18)	0.35581 (17)	0.0387 (5)	
C20	0.0436 (3)	0.3987 (2)	0.2929 (2)	0.0516 (6)	
H20	−0.0242	0.3298	0.2481	0.062*	
C21	0.0160 (4)	0.4993 (2)	0.2966 (2)	0.0644 (8)	
H21	−0.0702	0.4977	0.2541	0.077*	
C22	0.1154 (4)	0.6011 (2)	0.3627 (2)	0.0652 (8)	
H22	0.0971	0.6684	0.3641	0.078*	
C23	0.2412 (4)	0.6044 (2)	0.4266 (2)	0.0614 (7)	
H23	0.3073	0.6736	0.4719	0.074*	
C24	0.2703 (3)	0.50456 (19)	0.42393 (19)	0.0496 (6)	
H24	0.3557	0.5070	0.4678	0.060*	
C25	0.5679 (5)	0.6853 (5)	0.2243 (3)	0.145 (2)	
H25A	0.5795	0.7050	0.1586	0.217*	
H25B	0.5336	0.7397	0.2742	0.217*	
H25C	0.4915	0.6106	0.2105	0.217*	
C26	0.7191 (4)	0.6861 (2)	0.2698 (2)	0.0583 (7)	
C27	0.7140 (4)	0.6509 (3)	0.3687 (3)	0.0836 (10)	
H27A	0.8184	0.6597	0.3968	0.125*	
H27B	0.6489	0.5724	0.3527	0.125*	
H27C	0.6714	0.6975	0.4208	0.125*	

### Atomic displacement parameters (Å^2^)

**Table:**

	*U*^11^	*U*^22^	*U*^33^	*U*^12^	*U*^13^	*U*^23^
Ni1	0.03109 (17)	0.04030 (18)	0.02937 (16)	0.01737 (13)	0.00755 (12)	0.00847 (12)
Cl1	0.0403 (3)	0.0461 (3)	0.0298 (3)	0.0176 (2)	0.0057 (2)	0.0091 (2)
Cl2	0.0343 (3)	0.0692 (4)	0.0458 (3)	0.0239 (3)	0.0149 (3)	0.0207 (3)
O1	0.0746 (13)	0.0499 (10)	0.0325 (8)	0.0323 (9)	−0.0012 (8)	0.0069 (7)
O2	0.0356 (10)	0.0444 (9)	0.0774 (12)	0.0109 (8)	0.0200 (9)	0.0219 (8)
O3	0.0952 (19)	0.0966 (17)	0.1112 (19)	0.0373 (14)	0.0510 (16)	0.0313 (14)
N1	0.0432 (11)	0.0461 (10)	0.0289 (10)	0.0238 (9)	0.0058 (8)	0.0106 (8)
N2	0.0323 (10)	0.0534 (11)	0.0329 (10)	0.0193 (9)	0.0071 (8)	0.0139 (8)
N3	0.0315 (10)	0.0380 (10)	0.0407 (10)	0.0110 (8)	0.0092 (8)	0.0119 (8)
N4	0.0311 (10)	0.0380 (10)	0.0303 (9)	0.0151 (8)	0.0043 (7)	0.0081 (7)
C1	0.0470 (15)	0.0607 (15)	0.0455 (14)	0.0250 (12)	0.0089 (11)	0.0179 (12)
C2	0.0610 (18)	0.0815 (19)	0.0443 (15)	0.0328 (15)	0.0056 (13)	0.0294 (14)
C3	0.0678 (19)	0.093 (2)	0.0307 (13)	0.0324 (16)	0.0005 (12)	0.0163 (14)
C4	0.0563 (17)	0.0718 (17)	0.0315 (12)	0.0300 (14)	0.0017 (11)	0.0081 (12)
C5	0.0318 (12)	0.0545 (14)	0.0316 (12)	0.0171 (11)	0.0052 (9)	0.0085 (10)
C6	0.0321 (12)	0.0482 (13)	0.0306 (11)	0.0163 (10)	0.0076 (9)	0.0081 (10)
C7	0.0412 (14)	0.0467 (13)	0.0305 (12)	0.0147 (11)	−0.0003 (10)	0.0016 (10)
C8	0.0563 (17)	0.0578 (17)	0.0501 (15)	0.0099 (13)	0.0131 (13)	0.0068 (12)
C9	0.084 (2)	0.0482 (17)	0.069 (2)	0.0041 (15)	0.0085 (17)	0.0073 (14)
C10	0.109 (3)	0.0476 (16)	0.0538 (17)	0.0278 (17)	0.0028 (17)	−0.0041 (13)
C11	0.094 (2)	0.0696 (19)	0.0435 (15)	0.0437 (18)	0.0186 (15)	0.0057 (13)
C12	0.0602 (17)	0.0531 (15)	0.0385 (13)	0.0252 (13)	0.0129 (12)	0.0076 (11)
C13	0.0404 (13)	0.0410 (12)	0.0396 (12)	0.0156 (11)	0.0067 (10)	0.0130 (10)
C14	0.0387 (14)	0.0510 (14)	0.0494 (14)	0.0098 (11)	0.0110 (11)	0.0198 (11)
C15	0.0319 (13)	0.0618 (15)	0.0475 (14)	0.0174 (11)	0.0137 (11)	0.0165 (12)
C16	0.0348 (13)	0.0499 (14)	0.0420 (13)	0.0215 (11)	0.0093 (10)	0.0088 (10)
C17	0.0311 (12)	0.0379 (11)	0.0268 (10)	0.0131 (9)	0.0026 (9)	0.0064 (8)
C18	0.0328 (12)	0.0382 (11)	0.0319 (11)	0.0141 (10)	0.0039 (9)	0.0053 (9)
C19	0.0379 (13)	0.0387 (12)	0.0421 (12)	0.0174 (10)	0.0123 (10)	0.0098 (10)
C20	0.0562 (16)	0.0506 (14)	0.0491 (14)	0.0266 (13)	−0.0020 (12)	0.0073 (11)
C21	0.077 (2)	0.0723 (19)	0.0619 (18)	0.0486 (17)	0.0068 (15)	0.0216 (15)
C22	0.086 (2)	0.0531 (17)	0.0743 (19)	0.0423 (16)	0.0262 (17)	0.0230 (15)
C23	0.0629 (19)	0.0396 (14)	0.0752 (19)	0.0170 (13)	0.0205 (15)	0.0050 (13)
C24	0.0458 (15)	0.0444 (14)	0.0553 (15)	0.0162 (11)	0.0096 (12)	0.0077 (11)
C25	0.119 (4)	0.261 (6)	0.089 (3)	0.113 (4)	0.001 (3)	0.051 (3)
C26	0.0649 (19)	0.0529 (16)	0.0536 (16)	0.0272 (14)	0.0089 (15)	0.0010 (12)
C27	0.090 (3)	0.101 (2)	0.073 (2)	0.049 (2)	0.0144 (18)	0.0263 (18)

### Geometric parameters (Å, °)

**Table:**

Ni1—N3	2.0344 (18)	C10—H10	0.9300
Ni1—N1	2.0418 (18)	C11—C12	1.384 (3)
Ni1—N4	2.0879 (17)	C11—H11	0.9300
Ni1—N2	2.1188 (17)	C12—H12	0.9300
Ni1—Cl1	2.3944 (6)	C13—C14	1.379 (3)
Ni1—Cl2	2.4153 (7)	C13—H13	0.9300
O1—N1	1.373 (2)	C14—C15	1.370 (3)
O1—H1	0.8200	C14—H14	0.9300
O2—N3	1.383 (2)	C15—C16	1.381 (3)
O2—H2	0.8200	C15—H15	0.9300
O3—C26	1.200 (3)	C16—C17	1.382 (3)
N1—C6	1.290 (3)	C16—H16	0.9300
N2—C1	1.328 (3)	C17—C18	1.486 (3)
N2—C5	1.352 (3)	C18—C19	1.477 (3)
N3—C18	1.292 (3)	C19—C20	1.386 (3)
N4—C13	1.335 (3)	C19—C24	1.393 (3)
N4—C17	1.356 (3)	C20—C21	1.386 (3)
C1—C2	1.389 (3)	C20—H20	0.9300
C1—H1A	0.9300	C21—C22	1.371 (4)
C2—C3	1.363 (4)	C21—H21	0.9300
C2—H2A	0.9300	C22—C23	1.367 (4)
C3—C4	1.381 (4)	C22—H22	0.9300
C3—H3	0.9300	C23—C24	1.386 (3)
C4—C5	1.392 (3)	C23—H23	0.9300
C4—H4	0.9300	C24—H24	0.9300
C5—C6	1.488 (3)	C25—C26	1.464 (5)
C6—C7	1.477 (3)	C25—H25A	0.9600
C7—C12	1.387 (3)	C25—H25B	0.9600
C7—C8	1.392 (3)	C25—H25C	0.9600
C8—C9	1.380 (4)	C26—C27	1.477 (4)
C8—H8	0.9300	C27—H27A	0.9600
C9—C10	1.382 (4)	C27—H27B	0.9600
C9—H9	0.9300	C27—H27C	0.9600
C10—C11	1.365 (4)		
			
N3—Ni1—N1	170.18 (7)	C9—C10—H10	120.1
N3—Ni1—N4	76.84 (7)	C10—C11—C12	120.4 (3)
N1—Ni1—N4	99.25 (7)	C10—C11—H11	119.8
N3—Ni1—N2	94.03 (7)	C12—C11—H11	119.8
N1—Ni1—N2	76.92 (7)	C11—C12—C7	120.3 (2)
N4—Ni1—N2	91.16 (7)	C11—C12—H12	119.9
N3—Ni1—Cl1	99.43 (5)	C7—C12—H12	119.9
N1—Ni1—Cl1	89.47 (5)	N4—C13—C14	122.8 (2)
N4—Ni1—Cl1	89.49 (5)	N4—C13—H13	118.6
N2—Ni1—Cl1	166.31 (5)	C14—C13—H13	118.6
N3—Ni1—Cl2	87.94 (5)	C15—C14—C13	119.2 (2)
N1—Ni1—Cl2	95.62 (6)	C15—C14—H14	120.4
N4—Ni1—Cl2	164.76 (5)	C13—C14—H14	120.4
N2—Ni1—Cl2	88.97 (5)	C14—C15—C16	119.0 (2)
Cl1—Ni1—Cl2	93.97 (2)	C14—C15—H15	120.5
N1—O1—H1	109.5	C16—C15—H15	120.5
N3—O2—H2	109.5	C15—C16—C17	119.2 (2)
C6—N1—O1	115.73 (17)	C15—C16—H16	120.4
C6—N1—Ni1	120.35 (15)	C17—C16—H16	120.4
O1—N1—Ni1	123.85 (12)	N4—C17—C16	121.86 (19)
C1—N2—C5	118.66 (19)	N4—C17—C18	115.20 (18)
C1—N2—Ni1	127.91 (16)	C16—C17—C18	122.9 (2)
C5—N2—Ni1	113.37 (14)	N3—C18—C19	125.41 (19)
C18—N3—O2	116.43 (18)	N3—C18—C17	112.33 (19)
C18—N3—Ni1	120.22 (14)	C19—C18—C17	122.25 (18)
O2—N3—Ni1	122.03 (13)	C20—C19—C24	118.9 (2)
C13—N4—C17	117.97 (18)	C20—C19—C18	120.0 (2)
C13—N4—Ni1	127.23 (15)	C24—C19—C18	121.1 (2)
C17—N4—Ni1	114.43 (13)	C19—C20—C21	120.2 (2)
N2—C1—C2	123.1 (2)	C19—C20—H20	119.9
N2—C1—H1A	118.4	C21—C20—H20	119.9
C2—C1—H1A	118.4	C22—C21—C20	120.2 (3)
C3—C2—C1	118.1 (2)	C22—C21—H21	119.9
C3—C2—H2A	121.0	C20—C21—H21	119.9
C1—C2—H2A	121.0	C23—C22—C21	120.4 (3)
C2—C3—C4	120.1 (2)	C23—C22—H22	119.8
C2—C3—H3	119.9	C21—C22—H22	119.8
C4—C3—H3	119.9	C22—C23—C24	120.1 (3)
C3—C4—C5	118.7 (2)	C22—C23—H23	119.9
C3—C4—H4	120.6	C24—C23—H23	119.9
C5—C4—H4	120.6	C23—C24—C19	120.2 (2)
N2—C5—C4	121.2 (2)	C23—C24—H24	119.9
N2—C5—C6	116.14 (18)	C19—C24—H24	119.9
C4—C5—C6	122.6 (2)	C26—C25—H25A	109.5
N1—C6—C7	125.2 (2)	C26—C25—H25B	109.5
N1—C6—C5	113.03 (19)	H25A—C25—H25B	109.5
C7—C6—C5	121.78 (18)	C26—C25—H25C	109.5
C12—C7—C8	119.1 (2)	H25A—C25—H25C	109.5
C12—C7—C6	120.7 (2)	H25B—C25—H25C	109.5
C8—C7—C6	120.2 (2)	O3—C26—C25	123.1 (3)
C9—C8—C7	119.8 (3)	O3—C26—C27	122.5 (3)
C9—C8—H8	120.1	C25—C26—C27	114.4 (3)
C7—C8—H8	120.1	C26—C27—H27A	109.5
C8—C9—C10	120.7 (3)	C26—C27—H27B	109.5
C8—C9—H9	119.7	H27A—C27—H27B	109.5
C10—C9—H9	119.7	C26—C27—H27C	109.5
C11—C10—C9	119.7 (3)	H27A—C27—H27C	109.5
C11—C10—H10	120.1	H27B—C27—H27C	109.5
			
N3—Ni1—N1—C6	26.9 (5)	O1—N1—C6—C7	0.3 (3)
N4—Ni1—N1—C6	92.66 (17)	Ni1—N1—C6—C7	177.24 (16)
N2—Ni1—N1—C6	3.62 (17)	O1—N1—C6—C5	−179.56 (17)
Cl1—Ni1—N1—C6	−177.94 (17)	Ni1—N1—C6—C5	−2.7 (3)
Cl2—Ni1—N1—C6	−84.00 (17)	N2—C5—C6—N1	−0.9 (3)
N3—Ni1—N1—O1	−156.4 (4)	C4—C5—C6—N1	179.0 (2)
N4—Ni1—N1—O1	−90.70 (17)	N2—C5—C6—C7	179.2 (2)
N2—Ni1—N1—O1	−179.74 (18)	C4—C5—C6—C7	−0.9 (3)
Cl1—Ni1—N1—O1	−1.30 (16)	N1—C6—C7—C12	−121.8 (3)
Cl2—Ni1—N1—O1	92.64 (16)	C5—C6—C7—C12	58.1 (3)
N3—Ni1—N2—C1	3.12 (19)	N1—C6—C7—C8	58.4 (3)
N1—Ni1—N2—C1	179.2 (2)	C5—C6—C7—C8	−121.7 (2)
N4—Ni1—N2—C1	80.01 (19)	C12—C7—C8—C9	0.2 (4)
Cl1—Ni1—N2—C1	172.62 (17)	C6—C7—C8—C9	−179.9 (2)
Cl2—Ni1—N2—C1	−84.74 (18)	C7—C8—C9—C10	−0.4 (5)
N3—Ni1—N2—C5	−179.93 (15)	C8—C9—C10—C11	0.6 (5)
N1—Ni1—N2—C5	−3.81 (14)	C9—C10—C11—C12	−0.5 (5)
N4—Ni1—N2—C5	−103.04 (15)	C10—C11—C12—C7	0.3 (4)
Cl1—Ni1—N2—C5	−10.4 (3)	C8—C7—C12—C11	−0.2 (4)
Cl2—Ni1—N2—C5	92.21 (14)	C6—C7—C12—C11	−180.0 (2)
N1—Ni1—N3—C18	59.8 (5)	C17—N4—C13—C14	0.4 (3)
N4—Ni1—N3—C18	−7.67 (16)	Ni1—N4—C13—C14	172.90 (16)
N2—Ni1—N3—C18	82.58 (17)	N4—C13—C14—C15	0.3 (3)
Cl1—Ni1—N3—C18	−94.92 (16)	C13—C14—C15—C16	−0.4 (3)
Cl2—Ni1—N3—C18	171.40 (16)	C14—C15—C16—C17	−0.1 (3)
N1—Ni1—N3—O2	−106.6 (4)	C13—N4—C17—C16	−0.9 (3)
N4—Ni1—N3—O2	−174.12 (17)	Ni1—N4—C17—C16	−174.40 (16)
N2—Ni1—N3—O2	−83.87 (16)	C13—N4—C17—C18	177.00 (17)
Cl1—Ni1—N3—O2	98.64 (15)	Ni1—N4—C17—C18	3.5 (2)
Cl2—Ni1—N3—O2	4.95 (15)	C15—C16—C17—N4	0.8 (3)
N3—Ni1—N4—C13	−171.15 (18)	C15—C16—C17—C18	−176.94 (19)
N1—Ni1—N4—C13	18.03 (18)	O2—N3—C18—C19	−0.9 (3)
N2—Ni1—N4—C13	94.97 (17)	Ni1—N3—C18—C19	−168.04 (16)
Cl1—Ni1—N4—C13	−71.35 (16)	O2—N3—C18—C17	178.61 (16)
Cl2—Ni1—N4—C13	−174.69 (13)	Ni1—N3—C18—C17	11.4 (2)
N3—Ni1—N4—C17	1.61 (13)	N4—C17—C18—N3	−9.5 (3)
N1—Ni1—N4—C17	−169.20 (13)	C16—C17—C18—N3	168.4 (2)
N2—Ni1—N4—C17	−92.26 (14)	N4—C17—C18—C19	170.02 (18)
Cl1—Ni1—N4—C17	101.42 (13)	C16—C17—C18—C19	−12.1 (3)
Cl2—Ni1—N4—C17	−1.9 (3)	N3—C18—C19—C20	127.1 (3)
C5—N2—C1—C2	−1.4 (3)	C17—C18—C19—C20	−52.4 (3)
Ni1—N2—C1—C2	175.42 (19)	N3—C18—C19—C24	−51.9 (3)
N2—C1—C2—C3	0.2 (4)	C17—C18—C19—C24	128.6 (2)
C1—C2—C3—C4	1.3 (4)	C24—C19—C20—C21	1.3 (4)
C2—C3—C4—C5	−1.6 (4)	C18—C19—C20—C21	−177.7 (2)
C1—N2—C5—C4	1.1 (3)	C19—C20—C21—C22	−0.2 (4)
Ni1—N2—C5—C4	−176.19 (18)	C20—C21—C22—C23	−0.9 (4)
C1—N2—C5—C6	−178.99 (19)	C21—C22—C23—C24	0.8 (4)
Ni1—N2—C5—C6	3.7 (2)	C22—C23—C24—C19	0.3 (4)
C3—C4—C5—N2	0.4 (4)	C20—C19—C24—C23	−1.4 (4)
C3—C4—C5—C6	−179.5 (2)	C18—C19—C24—C23	177.6 (2)

### Hydrogen-bond geometry (Å, °)

**Table:**

*D*—H···*A*	*D*—H	H···*A*	*D*···*A*	*D*—H···*A*
O2—H2···Cl2	0.82	2.27	2.9582 (18)	142
O1—H1···Cl1^i^	0.82	2.91	3.4612 (16)	127
O1—H1···Cl1	0.82	2.37	3.0542 (16)	141

Symmetry codes: (i) −*x*+1, −*y*, −*z*+1.
